# Advancing inclusive development through community-based rehabilitation: a systematic literature review

**DOI:** 10.3389/fresc.2026.1824043

**Published:** 2026-07-21

**Authors:** Udeme Samuel Jacob, Mbulaheni Obert Maguvhe

**Affiliations:** Department of Inclusive Education, School of Educational Studies, College of Education, University of South Africa, South Africa

**Keywords:** community-based rehabilitation (CBR), empowerment, persons with disabilities, social inclusion, systematic review

## Abstract

**Background/purpose:**

Community-Based Rehabilitation (CBR) has become a widely adopted strategy for promoting the participation, empowerment, and well-being of people with disabilities, particularly in low- and middle-income contexts. Despite its global implementation, empirical evidence on the effectiveness of CBR interventions remains dispersed across disciplines. This study synthesises existing empirical research to examine the outcomes of CBR programmes and empowerment-oriented interventions for persons with disabilities.

**Methods:**

A systematic literature review was conducted in accordance with established review procedures and PRISMA reporting guidelines. Peer-reviewed studies were identified through database searches using predefined keywords related to disability, community-based rehabilitation, and empowerment. Studies were included if they reported empirical findings on CBR outcomes among persons with disabilities. Data from eligible studies were extracted and analysed to compare study characteristics, research designs, analytical approaches, and reported outcomes.

**Results:**

Nine empirical studies met the inclusion criteria. The studies employed diverse methodological approaches, including cross-sectional surveys, mixed-method designs, qualitative investigations, and quasi-experimental analyses with comparison groups. Across the studies, participation in CBR programmes was consistently associated with improvements in social inclusion, empowerment, participation, and access to services. Quasi-experimental studies provided stronger evidence of programme effectiveness, while qualitative and mixed-method studies highlighted experiential dimensions of empowerment and community engagement.

**Conclusion:**

The evidence indicates that CBR programmes contribute to improved participation and empowerment among persons with disabilities. Strengthening evaluation designs and expanding outcome measurement across all domains of the CBR matrix will enhance future research and support the development of inclusive community rehabilitation strategies.

## Introduction

Disability remains one of the most significant yet persistently marginalised dimensions of global inequality. The World Health Organisation and the World Bank estimate that approximately 15 per cent of the global population, representing more than one billion individuals, live with some form of disability (1–4). Prevalence rates vary across contexts. Low-income countries report higher rates than high-income nations, reflecting disparities in health systems, environmental risk exposure, and socioeconomic conditions (1, 4, 5). Women, older adults, and children experience disproportionate vulnerability due to intersecting social and developmental risks (1, 3, 4).

Disability extends beyond prevalence figures and intersects deeply with structural inequality. Poverty both contributes to and results from disability, reinforcing cycles of exclusion and restricted opportunity (5, 6). Individuals with disabilities frequently encounter discrimination, stigma, and systemic marginalisation that limit access to healthcare, education, employment, and civic participation (7–10). Structural barriers within transport systems, housing design, and workplace environments further constrain mobility and independence (9, 11, 12). These conditions demonstrate that disability is not solely an individual impairment, but a socially constructed experience shaped by institutional, environmental, and attitudinal barriers (13, 14).

The conceptual understanding of disability has evolved significantly. The medical model framed disability primarily as a deficit to be corrected or treated (15, 16). This perspective prioritised biomedical intervention and professional authority while often neglecting lived experience. The social model reoriented the discourse by identifying societal barriers as the principal source of disability-related disadvantage (17–19). Building on this foundation, the rights-based approach emphasised dignity, autonomy, equality, and full participation (20, 21). The United Nations Convention on the Rights of Persons with Disabilities formalised this normative shift and required states to address systemic exclusion through legal and policy reform (22, 23).

Despite these advances, many systems continue to operate within residual charity-oriented or institutional paradigms. Traditional welfare approaches often reinforce dependency and position persons with disabilities as passive beneficiaries of assistance (24, 25). Such models may unintentionally silence agency and restrict participation in decision-making processes (26). Scholars and practitioners increasingly advocate for empowerment and resilience frameworks that centre autonomy and capability development. Independent living philosophies prioritise skill acquisition and community integration (27). Technological innovations expand opportunities for participation and accessibility (28). Resilience frameworks in rehabilitation emphasise self-efficacy, adaptability, and resource mobilisation as essential capacities for navigating structural barriers (29). Educational and community-based empowerment initiatives further illustrate how structured capability-building enhances confidence and social engagement (30).

This transformation aligns with broader global development commitments. The Sustainable Development Goals underscore the need to reduce inequality and promote inclusive participation (31, 32). Inclusive development theory similarly asserts that sustainable progress requires equity, social justice, and participatory governance (33, 34). South–South cooperation models demonstrate how locally grounded empowerment strategies can strengthen institutional capacity in the Global South (35).

Within this evolving landscape, Community-Based Rehabilitation has emerged as a central strategy for operationalising empowerment at the community level. Community-Based Rehabilitation integrates health, education, livelihood, social participation, and empowerment components through locally coordinated interventions. Although CBR has been the subject of several previous reviews, the rationale for a contemporary synthesis remains compelling, as important evidence gaps persist. Existing reviews have often concentrated on specific impairment groups, selected intervention types, or narrowly defined outcomes such as functional status or quality of life. Some reviews focused primarily on methodological guidance for evaluating CBR rather than synthesising substantive programme outcomes. Others were conducted before the publication of more recent quasi-experimental and mixed-method studies that now expand the available evidence base. Consequently, current understanding remains fragmented regarding which domains of the CBR matrix receive the strongest empirical support, how findings vary across methodological designs, and the extent to which newer evidence strengthens confidence in reported outcomes (36–40), while others emphasise the need for stronger evaluation frameworks and more rigorous study designs (41). Service provision for children and individuals with complex needs in low- and middle-income countries remains uneven and under-resourced (42, 43). Digital innovations offer emerging opportunities to strengthen implementation, although systematic synthesis of such approaches remains limited (44).

A systematic review is needed to consolidate evidence on how Community-Based Rehabilitation and empowerment models foster strength, resilience, dignity, and sustainable inclusion. By integrating research from health sciences, education, social policy, and development studies, this review clarifies which approaches are effective, under what conditions, and for whom, particularly within structurally constrained contexts (38, 45). Such synthesis is essential for informing policy, strengthening implementation, and advancing the transition from care-based systems to capability-oriented inclusion.

This review differs from previous reviews in three ways. First, it synthesises contemporary empirical studies published between 2010 and 2025 within a rights-based and inclusive development context. Second, it compares findings across diverse methodological designs, including quasi-experimental, mixed-method, and quantitative studies, to examine how evidential strength varies by research design. Third, it maps reported outcomes across the domains of the CBR matrix rather than limiting attention to single indicators such as health status or quality of life. By doing so, the review provides a broader and more policy-relevant account of how CBR contributes to inclusion, participation, and empowerment.

Accordingly, this systematic review was guided by the following research questions:

What are the key characteristics of empirical studies evaluating Community-Based Rehabilitation programmes for persons with disabilities, including sample profiles, populations, settings, and study designs?
What methodological and analytical approaches have been used to evaluate CBR programmes, and how strong is the internal validity of the available evidence?
Which outcome domains within the Community-Based Rehabilitation matrix (health, education, livelihood, social inclusion, empowerment, and participation) have been examined most frequently and most comprehensively?
What is the reported direction and relative strength of findings on CBR outcomes across included studies?
What overall patterns, gaps, and priorities emerge from the evidence base to guide future research, policy, and practice in inclusive community development?

## Methods

### Review design and reporting

This study employed a systematic review to synthesise empirical evidence on the effectiveness of Community-Based Rehabilitation (CBR) and empowerment-oriented models for person with disabilities. The review was conducted and reported in accordance with the PRISMA 2020 statement to ensure transparency, reproducibility, and methodological rigour in the identification, screening, eligibility assessment, and synthesis processes (46–50).

A systematic design was appropriate given the interdisciplinary and methodologically heterogeneous nature of CBR research, which spans rehabilitation science, public health, education, and development studies. The objective was not to generate pooled effect sizes but to evaluate methodological rigour, domain coverage, and consistency in the reporting of effects across empirical evaluations. Given variability in study design, outcome operationalisation, and analytical strategies, a structured comparative narrative synthesis was adopted rather than meta-analytic aggregation.

### Data sources and search strategy

A comprehensive search was conducted in Scopus, selected for its extensive coverage of peer-reviewed journals across disability research, health sciences, social policy, and development scholarship. Scopus was also chosen because it provides robust citation indexing, advanced Boolean search capabilities, and broad international journal coverage, making it suitable for capturing interdisciplinary CBR literature within a single searchable platform. The search strategy was developed iteratively to capture empirical evaluations of CBR interventions and empowerment-oriented community models.

Search terms combined controlled vocabulary and keyword variations using Boolean operators and included:

“Community-Based Rehabilitation” OR “CBR” AND “persons with disabilities” OR “disability” AND “empowerment” OR “resilience” OR “participation” OR “livelihood” OR “quality of life” AND “effectiveness” OR “impact” OR “evaluation.”

Search was applied to titles, abstracts, and keywords to maximise sensitivity while maintaining conceptual specificity. The timeframe was restricted to publications between 2010 and 2025 to reflect contemporary rights-based and inclusive development frameworks. Only English-language, peer-reviewed journal articles were included to ensure comparability in methodological reporting standards. The database search yielded 650 records. No additional records were identified through registers or supplementary searches.

### Eligibility criteria

Eligibility criteria were defined as *a priori* and applied before the screening stage. Studies were eligible for inclusion if they:

1. Evaluated Community-Based Rehabilitation or comparable community-level empowerment interventions;
2. Included people with disabilities as the primary participant group;
3. Reported measurable outcomes aligned with one or more domains of the WHO CBR matrix (health, education, livelihood, social inclusion, empowerment, or participation);
4. Used quantitative, quasi-experimental, mixed-method designs with extractable quantitative findings, or meta-analytic approaches.

Studies were excluded if they were conceptual papers, policy commentaries, editorials, conference abstracts, dissertations, grey literature, or qualitative-only investigations without measurable outcome indicators. Excluding qualitative-only studies was necessary to maintain analytical comparability in terms of effect direction and inferential strength. Previously published systematic reviews and meta-analyses were not included as primary studies in the comparative synthesis; however, they were retained for background framing and citation tracking where relevant.

### Eligibility criteria

Studies were eligible for inclusion if they evaluated Community-Based Rehabilitation or comparable community-level empowerment interventions and included persons with disabilities as primary participants. Eligible studies were required to report measurable outcomes aligned with one or more domains of the WHO CBR matrix, including health, education, livelihood, social inclusion, or empowerment. Acceptable study designs included quantitative, quasi-experimental, mixed-method (with measurable quantitative components), and meta-analytic approaches.

Studies were excluded if they were purely conceptual, policy commentaries, editorials, conference abstracts, grey literature, or qualitative-only investigations lacking measurable outcomes. Excluding qualitative-only studies was necessary to maintain analytical comparability in terms of effect direction and inferential strength.

### Data extraction

Data were extracted using a structured and standardised template to ensure consistency across studies. Extracted variables included author(s) and year of publication; sample size and participant characteristics; study design; duration of intervention or follow-up; analytical methods; outcome domains aligned with the CBR matrix; effect direction and magnitude; and indicators of internal validity. Extraction prioritised analytical findings and inferential claims rather than descriptive contextual information. Where reported, effect size estimates and statistical significance indicators were documented. Data extraction was conducted iteratively to ensure accuracy and completeness. Data extraction was completed independently by two reviewers, and the extracted forms were then compared for accuracy, completeness, and consistency. Any discrepancies were resolved through discussion and verification against the source article.

### Analytical strategy

Based on methodological heterogeneity, a structured comparative synthesis approach was adopted. Analysis was organised into four components:

1. Study characteristics and methodological progression
2. Analytical design strength and internal validity
3. Outcome domain coverage across the CBR matrix
4. Effect direction and magnitude relative to methodological rigour

Emphasis was placed specifically on contrasting cross-sectional case-control studies with regression-based quasi-experimental designs employing Propensity Score Matching and Difference-in-Differences techniques. These methodological distinctions were evaluated to assess inferential robustness and causal credibility. Outcome domains were mapped to assess balance across health, education, livelihood, social inclusion, and empowerment components. Effect magnitude was interpreted relative to analytical rigour, allowing identification of patterns linking methodological strength to reported impact.

### Quality and bias considerations

Formal risk-of-bias scoring was not undertaken, consistent with systematic reviews synthesising heterogeneous quasi-experimental and meta-analytic evidence (49). Instead, methodological quality was assessed comparatively based on the presence of control groups, matching procedures, longitudinal design, transparency in statistical modelling, and reporting of effect sizes. Quality appraisal was conducted independently by two reviewers using these predefined criteria, with disagreements resolved through consensus discussion. Studies were not excluded solely on quality grounds; rather, appraisal findings informed the interpretation of evidential strength within the synthesis.

Potential selection bias arises from restricting inclusion to English-language, peer-reviewed articles indexed in Scopus. Publication bias and contextual heterogeneity may also influence generalisability. However, transparent eligibility criteria, systematic screening, and PRISMA-compliant reporting mitigate procedural bias and strengthen reproducibility.

## Results

### Study selection

**Figure 1:**
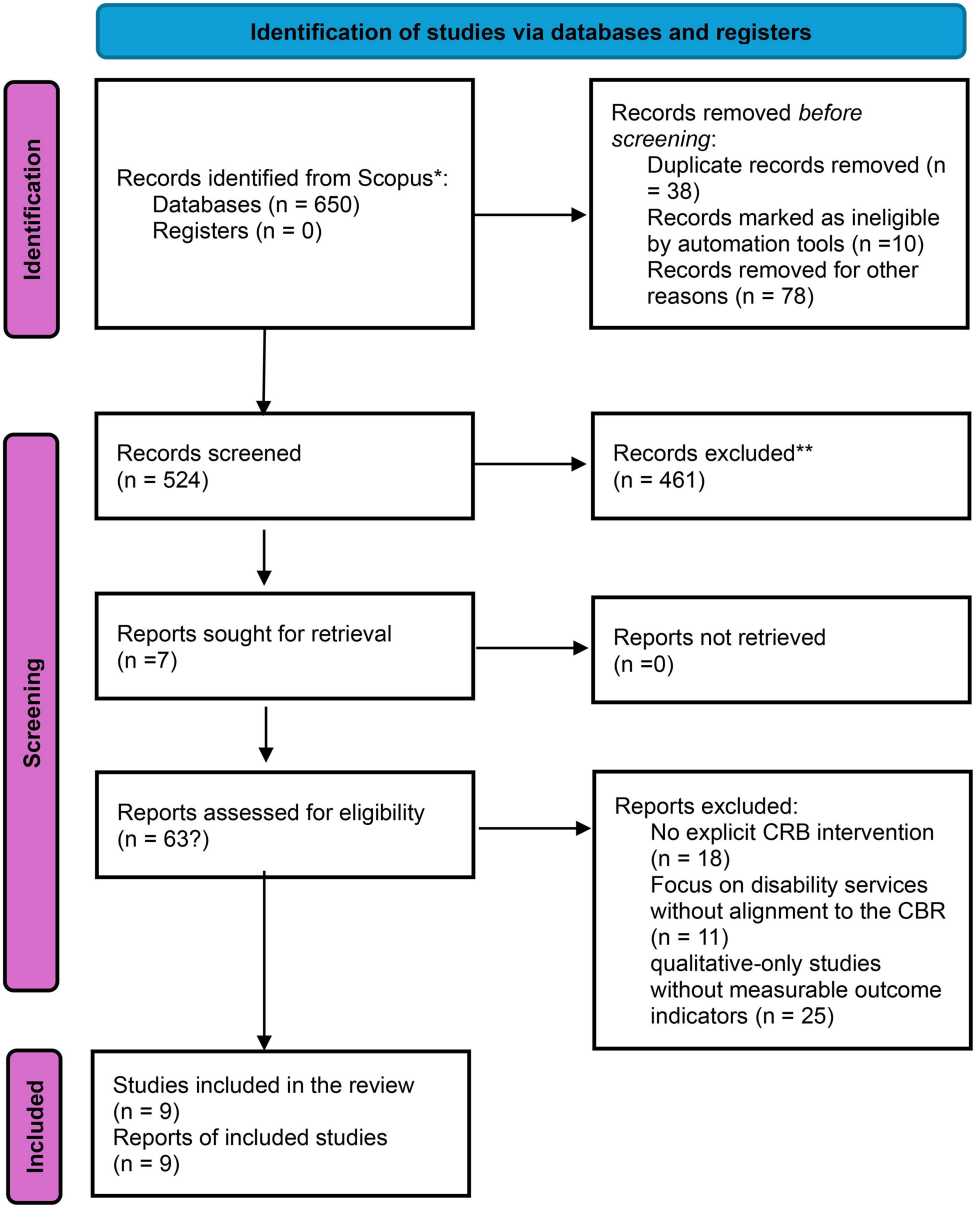
PRISMA 2020 flow diagram of the study selection process.

The study selection process is presented in Figure 1. A total of 650 records were identified through the database search. After removing 38 duplicate records, 612 records remained. A further 10 records were excluded via automated filtering, and 78 records were removed for other reasons, including non-article formats and indexing errors. This left 524 records for title and abstract screening.

During title and abstract screening, 461 records were excluded for not meeting eligibility criteria. Common reasons for exclusion included a lack of empirical evaluation, weak alignment with Community-Based Rehabilitation (CBR), a conceptual discussion only, or the absence of measurable outcomes. Sixty-three full-text articles were assessed for eligibility. Of these, 54 articles were excluded after full-text review, leaving 9 studies included in the final synthesis.

The full-text exclusions comprised studies without an explicit evaluation of CBR interventions, studies examining disability services that were not aligned with the CBR matrix, and qualitative-only investigations without measurable outcome indicators. The complete screening pathway is shown in Figure 1.

Table 1 summarises the characteristics of the nine studies included in the review. The studies were published between 2014 and 2025, indicating sustained scholarly attention to the effectiveness of community-based rehabilitation interventions for persons with disabilities.

**Table 1:** Characteristics of included studies.

Study	Sample Size	Population	Study Design	Duration
Biggeri et al. (57)	∼300+	Persons with disabilities	Case-control	Cross-sectional
Mauro et al. (56)	∼500+	Persons with disabilities	Quasi-experimental	Cross-sectional
Iemmi et al. (39)	Multi-study pooled sample	Persons with disabilities	Meta-analysis	Multi-study
Trani et al. (51)	1,861 intervention; 1,132 control	Persons with disabilities	Longitudinal quasi-experimental	36 months
Trani et al. (52)	2,639	Persons with disabilities	Quasi-experimental	Multi-wave
Asres (54)	222	Persons with disabilities	Mixed-method evaluation	Cross-sectional
Ginting et al. (58)	Community cohort	individuals with lifelong support needs	Mixed-method sequential explanatory	Cross-sectional
Mukami et al. (53)	148	Adults with disabilities	Mixed-method descriptive	Cross-sectional
Madsen et al. (55)	115 observation; 15 interviews	People with disabilities	Ethnographic qualitative study	5 months

Sample sizes varied widely across studies. Larger quantitative studies were reported by Trani et al. (51) and Trani et al. (52), involving over 1,800 and 2,600 participants, respectively, which enhances the statistical robustness of their findings. In contrast, smaller studies, such as those by Mukami et al. (53) and Asres (54), examined more focused participant groups to explore programme implementation and outcomes in specific contexts. Qualitative insights were provided by Madsen et al. (55), who employed ethnographic observation and interviews to examine empowerment experiences in outdoor rehabilitation settings.

Methodologically, the evidence base is highly diverse. Cross-sectional and quasi-experimental quantitative designs dominate, particularly in earlier studies such as Mauro et al. (56) and Biggeri et al. (57). More rigorous quasi-experimental approaches using matched comparison groups were applied in the studies by Trani et al., strengthening causal inference. Several recent studies also adopted mixed-method designs that combine quantitative outcomes with qualitative insights. The included studies focus primarily on persons with disabilities participating in CBR programmes, providing a diverse yet complementary evidence base for understanding programme effectiveness.

Table 2 summarises the analytical designs used in the included studies and the basis for the comparative appraisal of internal validity. Stronger ratings were assigned to studies that incorporated matched comparison groups, longitudinal follow-up, statistical adjustment, or evidence synthesis across multiple studies. Moderate ratings were assigned to studies that generated useful empirical evidence but lacked a robust counterfactual comparison. Lower ratings reflected designs that were primarily descriptive or qualitative and therefore less suited to causal inference.

**Table 2:** Design and analytical characteristics of included studies.

Study	Design Type	Comparison Group	Analytical Approach	Basis for Internal Validity Rating	Internal Validity
Biggeri et al. (57)	Case-control	Participants in CBR compared with non-participants	Multivariate regression	Comparative design with statistical adjustment; cross-sectional data	Moderate
Mauro et al. (56)	Quasi-experimental	The intervention group compared with the non-CBR comparison group	Multilevel modelling	Comparison group with multilevel adjustment; non-random allocation	Moderate–Strong
Iemmi et al. (39)	Meta-analysis	Multiple studies	Meta-analytic synthesis	Systematic aggregation of multiple studies; dependent on the included study quality	Strong
Trani et al. (51)	Longitudinal quasi-experimental	Matched controls	Propensity Score Matching and Difference-in-Difference	Matched controls, longitudinal design, temporal comparison	Strong
Trani et al. (52)	Quasi-experimental	Matched controls	Propensity Score Matching	Matching procedures to reduce selection bias	Strong
Asres (54)	Mixed-method evaluation	No	Descriptive statistics and thematic analysis	Multi-source data, but no counterfactual comparison	Moderate
Ginting et al. (58).	Mixed-method sequential explanatory	No	Integrated quantitative and qualitative analysis	Sequential mixed-method triangulation; no control group	Moderate
Mukami et al. (53)	Mixed-method descriptive	No	Descriptive statistics and thematic interpretation	Descriptive evidence with limited analytical control	Moderate–Weak
Madsen et al. (55)	Ethnographic qualitative	No	Interpretive description and thematic analysis	Rich contextual insight; no causal comparison design	Moderate

Across the evidence base, quasi-experimental studies by Trani et al. (51, 52) and the synthesis by Iemmi et al. (39) demonstrated the strongest analytical designs. Mixed-method and descriptive studies contributed contextual and implementation evidence, although with lower inferential certainty. Overall, the table indicates that the literature includes both higher-rigour comparative evaluations and practice-based studies that illuminate how CBR operates in diverse settings.

Table 3 maps reported outcomes against the key domains of the CBR matrix. Across the studies, the most frequently addressed outcomes were social inclusion, empowerment, and participation, indicating that CBR research increasingly focuses on broader social and capability-based outcomes rather than solely on clinical rehabilitation indicators.

**Table 3:** Outcome domains and community-based rehabilitation (CBR) matrix coverage.

Study	Health	Education	Livelihood	Social Inclusion	Empowerment	Participation
Biggeri et al. (57)	✓	✓	✓	✓	✓	✓
Mauro et al. (56)	✓	✓	Limited	✓	✓	✓
Iemmi et al. (39).	✓	Indirect	Limited	✓	Limited	Indirect
Trani et al. (51)	✓	✓	✓	✓	✓	✓
Trani et al. (52)	✓	✓	Limited	✓	✓	✓
Asres (54)	✓	Limited	Limited	✓	✓	Limited
Ginting et al. (58)	✓	✓	✓	✓	✓	✓
Mukami et al. (53)	✓	✓	Limited	✓	✓	✓
Madsen et al. (55)	Limited	Limited	Limited	✓	✓	✓

✓ = Directly measured and analysed.

Indirect=Examined through proxy indicators.

Limited=Mentioned but not robustly measured.

Health-related outcomes were also widely examined, particularly in studies evaluating quality of life, service access, or functional well-being. In contrast, livelihood and education outcomes appeared less consistently across the literature, with several studies reporting only limited or indirect measurement of these domains.

Notably, large quasi-experimental evaluations such as those conducted by Trani et al. and Mauro et al. tended to assess multiple CBR domains simultaneously, reflecting the multidimensional nature of CBR interventions. Mixed-method and qualitative studies, including Mukami et al. (53) and Madsen et al. (55) primarily emphasised empowerment, participation, and community connection. Overall, the table highlights that while the literature broadly aligns with the multidimensional CBR framework, coverage across domains remains uneven, with social participation and empowerment receiving the strongest empirical attention.

Table 4 summarises reported outcomes, measurement approaches, and the evidential basis of findings across the included studies. Because outcome measures, analytical models, and reporting formats varied substantially, direct comparisons of effect sizes across studies were not always possible. For this reason, the table emphasises reported statistical evidence, measurement indicators, and design strength rather than unsupported generic labels of impact size (49).

**Table 4:** Reported outcomes, measures, and evidential strength of findings .

Study	Primary Outcome Domain(s)	Outcome Measure(s)/Indicator(s)	Reported Statistical Evidence	Direction of Findings	Basis for Interpreting Strength of Evidence
Biggeri et al. (57)	Participation, wellbeing	Participation indicators, self-reported wellbeing, comparative programme outcomes	Adjusted group differences reported through multivariate regression	Favourable	Comparative design with regression adjustment; statistical associations reported
Mauro et al. (56)	Wellbeing, inclusion	Inclusion and well-being indicators across groups	Significant between-group differences were estimated using multilevel modelling	Favourable	Quasi-experimental design with multilevel analysis
Iemmi et al. (39)	Health, functioning	Pooled outcomes from multiple disability and rehabilitation studies	Meta-analytic synthesis reporting positive pooled effects across included outcomes	Favourable	Highest evidential level in review; multiple-study synthesis
Trani et al. (51)	Participation, social inclusion	Participation, inclusion, and longitudinal change indicators	Effects estimated using Propensity Score Matching + Difference-in-Differences	Favourable	Strong quasi-experimental design with matched controls over time
Trani et al. (52)	Service access, participation	Access to services and participation indicators	Adjusted differences estimated using Propensity Score Matching	Favourable	Strong comparative design with matching procedures
Asres (54)	Service access, empowerment	Service utilisation, empowerment, participant reports	Descriptive trends supported by mixed-method findings	Favourable	Useful applied evidence, but no formal counterfactual comparison
Ginting et al. (58).	Quality of life, empowerment	Quality of life indicators, empowerment experiences	Convergent quantitative and qualitative findings	Favourable	Mixed-method triangulation without control group
Mukami et al. (53)	Social inclusion, service access	Inclusion experiences, access indicators	Descriptive statistical patterns with thematic support	Favourable	Descriptive evidence with limited analytical control
Madsen et al. (55)	Empowerment, community engagement	Interview accounts, observational evidence, and experiential participation	Qualitative thematic evidence of perceived gains	Favourable	Contextual and mechanism-focused evidence rather than causal estimation

Across the included studies, findings were generally favourable, although the certainty of evidence differed by design and reporting quality. Stronger confidence was associated with quasi-experimental and evidence synthesis studies that used matched comparisons or pooled analyses (39, 51, 52). In contrast, mixed-method and qualitative studies primarily contributed contextual evidence regarding empowerment, participation, and programme experience (53–55, 58).

### Structured comparative synthesis

The included studies varied substantially in sample size, design, analytical strategy, and inferential strength. This variation is important because conclusions about Community-Based Rehabilitation (CBR) depend not only on whether outcomes were favourable, but also on how those outcomes were evaluated. Across the evidence base, a clear methodological gradient was evident: studies using stronger comparative designs generally provided more persuasive evidence than descriptive investigations, while qualitative work contributed explanatory insight into programme processes and participant experiences.

### Methodological strength and internal validity

The strongest evidence came from studies employing matched comparison groups, longitudinal follow-up, or advanced statistical adjustment. The quasi-experimental evaluations by Trani et al. (51) and Trani et al. (52), which used Propensity Score Matching and, in one case, Difference-in-Differences estimation, provided the highest level of confidence in the reported programme effects because they sought to reduce selection bias and baseline group differences. The evidence synthesis by Iemmi et al. (39) also strengthened the broader evidence base by integrating findings across multiple studies.

By contrast, cross-sectional, descriptive, and mixed-method studies offered more limited causal certainty. Their findings remained valuable, particularly for understanding implementation realities, local service delivery, and participant perspectives, but they were less able to determine whether observed improvements were directly attributable to programme participation. Positive findings should therefore be interpreted with greater confidence when supported by stronger comparative designs.

### Outcome domains across the CBR matrix

A second pattern concerned the distribution of outcomes across domains of the CBR matrix. Social inclusion, participation, and empowerment were the most consistently represented domains across studies. This suggests that the literature has focused strongly on community presence, agency, and social engagement as central indicators of programme value. Positive findings in these areas indicate that CBR may be particularly relevant for addressing relational and participation barriers experienced by persons with disabilities (51–54, 58).

Health-related outcomes were also commonly reported, especially in relation to well-being, functioning, or access to services. In contrast, education and livelihood outcomes received comparatively less attention and were often measured indirectly or only briefly. This imbalance limits the ability to determine whether CBR delivers equally strong benefits across all intended domains and highlights the need for broader future evaluations that better capture employment, educational progression, and longer-term socioeconomic outcomes.

### Direction and relative magnitude of findings

Despite methodological diversity, the direction of findings was notably consistent. Across quantitative, mixed-method, and qualitative studies, outcomes were generally reported as favourable. Stronger quasi-experimental studies identified improvements in participation, service access, and social inclusion, while descriptive and mixed-method studies more often highlighted empowerment, confidence, community engagement, and perceived inclusion (51, 52, 56, 57).

However, consistency in a positive direction should not be interpreted as proof of identical effect magnitude across studies. Variation in measures, intervention models, follow-up periods, and analytical methods means that direct comparison of impact size remained difficult. The evidence, therefore, supports cautious optimism rather than uniform conclusions about programme effectiveness.

### Overall synthesis

Taken together, the evidence suggests that CBR is a promising and contextually relevant strategy for advancing disability inclusion at the community level. The most convincing evidence emerged from studies with stronger internal validity, whereas smaller descriptive and qualitative studies added important contextual understanding of how programmes may foster empowerment and participation in everyday settings. At the same time, uneven domain coverage and methodological heterogeneity indicate that further rigorous and multidimensional evaluation remains necessary.

## Discussion

This systematic review synthesised empirical evidence on the effectiveness of Community-Based Rehabilitation (CBR) and empowerment-oriented interventions for persons with disabilities. Across the nine studies analysed, the literature consistently indicates that CBR programmes improve participation, social inclusion, empowerment, and access to services. Because the evidence base was methodologically diverse, these findings should be interpreted as broadly supportive rather than uniformly conclusive evidence of effectiveness. The overall pattern is consistent with contemporary understandings of disability that emphasise the interaction between individuals and their social environments rather than impairment alone (13, 14).

### Interpretation of evidence

One important implication of the review is that CBR appears most relevant when understood as a multidimensional inclusion strategy rather than a narrow rehabilitation service. Community-Based Rehabilitation was originally designed to integrate the domains of health, education, livelihood, social participation, and empowerment, thereby extending its value beyond that of clinical rehabilitation alone (36, 38, 39). Community-based approaches seek to address barriers related to participation, local support systems, social relationships, and access to opportunities.

The concentration of positive findings in empowerment and participation domains suggests that these community-facing outcomes may be among the most visible contributions of CBR programmes. This pattern was reflected across several included studies reporting gains in confidence, participation, inclusion, or service access (51–54, 58). However, this should not be interpreted to mean that all programme components are equally effective or equally evidenced across settings.

The review also indicated uneven coverage across the CBR matrix. Social inclusion, participation, and empowerment outcomes were more frequently examined than education and livelihood outcomes. Similar concerns regarding uneven outcome assessment and evaluation scope have been noted in earlier reviews of CBR research (36, 38).

This imbalance may reflect both research priorities and measurement challenges. Outcomes such as confidence, engagement, or perceived inclusion are often easier to observe within shorter programme periods than structural outcomes such as employment trajectories or sustained educational attainment. As a result, the present evidence base offers stronger insight into some domains than others.

Although the review prioritised studies with measurable outcomes, Madsen et al. (55) was retained because it examined empowerment within an explicitly community-based rehabilitation context and provided evidence on mechanisms through which participation and social engagement may occur. Its inclusion was not intended as equivalent causal evidence alongside quasi-experimental studies, but as contextual evidence that complemented quantitative findings. The study was therefore interpreted separately and given lower inferential weight when drawing overall conclusions. This distinction is important because empowerment and participation are central domains of the CBR framework and may not always be fully captured through standardised quantitative metrics.

### Alignment with rights-based disability frameworks

The reviewed studies also reinforce the shift from charity-oriented welfare models toward rights-based disability frameworks. Earlier welfare approaches often positioned persons with disabilities as passive recipients of support rather than active participants in social and economic life (24, 25). In contrast, contemporary rights-based perspectives emphasise autonomy, dignity, and participation in decision-making processes (20, 21).

The reviewed studies provide evidence that CBR programmes translate these principles into practical interventions at the community level. By promoting participation in social activities, improving access to services, and strengthening individual agencies, CBR programmes support the broader objectives of inclusive development. These outcomes align closely with the goals of the United Nations Convention on the Rights of Persons with Disabilities, which emphasises equal participation and social inclusion (22, 23).

### Methodological patterns in CBR evaluation

The evidence base also reveals important methodological patterns in the evaluation of CBR interventions. Studies employing quasi-experimental designs provide the strongest evidence regarding programme effectiveness. Evaluations conducted by Trani et al. (51, 52), for example, use matched comparison groups and advanced statistical techniques to estimate programme effects. These approaches strengthen causal inference by addressing potential selection bias and confounding variables.

The application of Propensity Score Matching and Difference-in-Difference estimation represents a methodological advancement in disability research. Scholars have previously emphasised the need for more rigorous evaluation frameworks for assessing rehabilitation interventions (36, 41). The presence of quasi-experimental studies within the current evidence base therefore increases confidence in reported programme outcomes.

Nevertheless, considerable methodological heterogeneity persists across studies. Several investigations rely on cross-sectional or descriptive designs that lack comparison groups. Studies conducted by Asres (54), Ginting et al. (58), and Mukami et al. (53) provide important contextual insights into programme implementation but offer limited causal inference. Earlier systematic reviews have similarly identified inconsistencies in evaluation frameworks and outcome measurement across CBR research (36, 38). These differences partly reflect the practical challenges associated with evaluating complex community-based interventions, particularly in resource-constrained contexts.

### Outcome domains within the CBR matrix

Another notable observation concerns the uneven distribution of outcome domains examined across the CBR matrix. Most studies focus on empowerment, participation, and social inclusion outcomes, whereas education and livelihood indicators appear less frequently in programme evaluations. This pattern reflects broader trends within disability research that prioritise participation and capability development.

However, the limited attention to livelihood and education outcomes highlights an important gap in programme evaluation. Inclusive development frameworks emphasise that sustainable disability inclusion requires improvements across multiple domains, including employment opportunities, education systems, and accessible health services (33, 34). Future research should therefore adopt more comprehensive evaluation frameworks that assess outcomes across all domains of the CBR matrix.

### Complementarity of quantitative and qualitative evidence

The literature reviewed also demonstrates the complementary value of quantitative and qualitative approaches in evaluating rehabilitation programmes. Quantitative studies provide statistical evidence on programme effectiveness and the magnitude of outcomes, whereas qualitative research offers insight into the mechanisms by which rehabilitation interventions influence empowerment and participation.

For example, the ethnographic research conducted by Madsen et al. (55) illustrates how rehabilitation activities embedded within community environments can promote empowerment through everyday social interaction. These insights support calls for mixed-method research designs that integrate statistical analysis with contextual understanding of programme implementation (36). Combining methodological approaches, therefore, enables a more comprehensive understanding of how CBR programmes operate in diverse social contexts.

### Limitations and directions for future research

Several limitations within the existing evidence base warrant attention. The predominance of cross-sectional designs limits causal inference in many studies. In addition, variations in outcome measurement make it difficult to compare findings across investigations. Many studies also focus on specific regional contexts, which may restrict the generalisability of findings to other cultural or institutional environments.

Future research should prioritise longitudinal and quasi-experimental designs that enable stronger causal inference regarding programme impacts. Researchers should also expand measurement of livelihood and educational outcomes to better capture the multidimensional goals of community-based rehabilitation. Emerging digital technologies may further enhance programme delivery and monitoring. Assistive technologies and digital platforms have been identified as promising tools for improving accessibility and participation among persons with disabilities (28). Continued investigation of technology-enabled CBR models may therefore strengthen the effectiveness and scalability of community-based rehabilitation initiatives.

The evidence synthesised in this review demonstrates that CBR programmes contribute meaningfully to participation, empowerment, and social inclusion among persons with disabilities. Although methodological variation remains across the literature, the consistency of positive outcomes across diverse study designs reinforces the broader contribution of community-based rehabilitation to inclusive community development.

## Conclusion

This systematic review examined empirical evidence on Community-Based Rehabilitation (CBR) and empowerment-oriented interventions for persons with disabilities. Overall, the reviewed studies suggested that CBR programmes were generally associated with positive outcomes in participation, social inclusion, empowerment, and access to services. Given the modest and methodologically heterogeneous evidence base, these findings should be interpreted as encouraging rather than definitive proof of effectiveness. The available evidence nevertheless indicates that community-level approaches may play an important role in addressing not only health-related needs, but also the social and structural barriers that restrict full participation.

The review also suggested that multidimensional approaches aligned with the CBR matrix may be more responsive to the complex realities of disability inclusion than narrowly focused interventions. The available evidence points to the potential value of CBR initiatives that integrate multiple domains of support, including health, social participation, and community engagement, although comparative effectiveness was not directly examined. Programmes that combine support for participation, access to local services, and empowerment appeared particularly promising across several contexts. However, outcome patterns varied across studies, and stronger confidence in programme impact was more evident in investigations using quasi-experimental methods or comparison groups than in descriptive designs alone.

Important gaps remain in the current literature. Many studies relied on cross-sectional or observational designs, and some domains of the CBR matrix, especially education and livelihood, were less frequently assessed than participation and empowerment outcomes. Future research should prioritise clearer intervention descriptions, stronger and more standardised outcome measures, broader domain coverage, and longer-term follow-up where feasible. Greater use of mixed-method and quasi-experimental designs may further strengthen the understanding of how CBR works across settings.

From a practical perspective, policymakers and service providers may consider investing in locally adaptable, cross-sector, and participation-oriented rehabilitation models while also strengthening monitoring and evaluation systems. Continued evidence development will be important for determining when, where, and for whom CBR offers the greatest benefit in advancing inclusive community development.
